# Robotic versus laparoscopic left colectomy: a propensity score matched analysis from a bi-centric experience

**DOI:** 10.1007/s11701-023-01634-7

**Published:** 2023-05-29

**Authors:** Leonardo Solaini, Giuseppe Giuliani, Davide Cavaliere, Antonio Bocchino, Michele Di Marino, Andrea Avanzolini, Andrea Coratti, Giorgio Ercolani

**Affiliations:** 1 Department of Medical and Surgical Sciences (DIMEC), University of Bologna, Bologna, Italy; 2 General and Oncologic Surgery, Morgagni Pierantoni Hospital, Ausl Romagna, Via c. Forlanini 34, Forlì, Italy; 3 Department of General and Emergency Surgery, Misericordia Hospital, Azienda Usl Toscana Sud Est, Grosseto, Italy

**Keywords:** Laparoscopy, Robot, Robot-assisted, Left emicolectomy, Diverticular disease, Colon cancer

## Abstract

The advantages of using the robotic platform may not be clearly evident in left colectomies, where the surgeon operates in an “open field” and does not routinely require intraoperative suturing. Current evidences are based on limited cohorts reporting conflicting outcomes regarding robotic left colectomies (RLC). The aim of this study is to report a bi-centric experience with robotic left colectomy in order to help in defining the role of the robotic approach for these procedures. This is a bi-centric propensity score matched study including patients who underwent RLC or laparoscopic left colectomy (LLC) between January 1, 2012 and May 1, 2022. RLC patients were matched to LLC patients in a 1:1 ratio. Main outcomes were conversion to open surgery and 30-day morbidity. In total, 300 patients were included. Of 143 (47.7%) RLC patients, 119 could be matched. After matching, conversion rate (4.2 vs. 7.6%, *p* = 0.265), 30-day morbidity (16.1 vs. 13.7%, *p* = 0.736), Clavien–Dindo grade ≥ 3 complications (2.4 vs 3.2%, *p* = 0.572), transfusions (0.8 vs. 4.0%, *p* = 0.219), and 30-day mortality (0.8 vs 0.8%, *p* = 1.000) were comparable for RLC and LLC, respectively. Median operative time was longer for RLC (296 min 260–340 vs. 245, 195–296, *p* < 0.0001). Early oral feeding, time to first flatus, and hospital stay were similar between groups. RLC has safety parameters as well as conversion to open surgery comparable with standard laparoscopy. Operative time is longer with the robotic approach.

## Introduction

The robotic system has been increasingly adopted in colorectal surgery since its introduction [1]. However, the robotic approach was mainly used for right colectomy and rectal resection. In right colectomy, the robotic platform has been associated with an increased rate of intra-corporeal anastomoses and with reduced conversion rates [2]. In rectal resection, the higher dexterity guaranteed by the Endowrist technology has been helping the surgeon in dissecting in a narrow field such as pelvis, allowing less conversions to open surgery and an improved quality of the mesorectal excision [3–5].

A recent meta-analysis showed that robotic left colectomy (RLC) has been associated with lower conversion and postoperative morbidity rates compared with the standard laparoscopic technique [6]. However, its outcome has been significantly influenced by two large studies [7, 8] based on national US databases representing more than the 95% of the weight of the pooled results. This, along with the conflicting results reported in the remaining included studies, might have limited the generalizability of the outcomes published in the meta-analysis.

In light of this, the aim of this study was to present data on RLC from two Italian centers in order to verify the validity of the results available in the current literature and to add useful data for future studies.

## Materials and methods

This study was performed according to the strengthening the reporting of cohort studies in surgery (STROCSS) guidelines [9]. The local ethics committee approved the study protocol.

### Design and patients

This is a retrospective cohort study, which was performed in two Italian centers. All consecutive patients who underwent robotic left colectomy between January 1, 2012 and May 1, 2022 were screened for inclusion. Included cases were compared with laparoscopic left colectomy (LLC) performed between May 1, 2017 and May 1, 2022. Patients were excluded if they had a synchronous resection for metastatic disease or urgent/emergency surgery. Patients were categorized according to the method of surgery: RLC or LLC.

### Variables and definitions

Baseline characteristics collected included sex, age, American Society of Anesthesiologists (ASA) score, Body Mass Index (BMI), Charlson comorbidity index (CCI) [10] indication for surgery, and the inclusion in an enhanced recovery program. Operative variables collected included operative time, conversion to open surgery, ligation technique (high tie versus low tie [11]), creation of stoma, harvested lymph nodes, and additional resection. Postoperative outcomes were postoperative complications and mortality, re-admissions within 30 days from discharge, time to first flatus, time to oral feeding, and length of hospital stay.

Complications were graded according to Dindo et al. [12].

Complications, re-admissions, and mortality were all collected up to 30 days postoperatively. All data were stored and processed anonymously.

### Operative technique

A medial to lateral approach was most frequently performed both in LLC and RLC and high tie performed in the procedures performed for malignant disease. The splenic flexure was mobilized according to patients’ anatomy and surgeons’ preference. Linear and circular stapling devices were routinely used to perform anastomoses via the double-stapling technique. Colorectal anastomosis was performed laparoscopically after un-docking. The da Vinci Si^®^ and Xi^®^ robotic platforms (Intuitive Surgical, Sunnyvale, CA, USA) were used.

All procedures were performed by expert surgeons in minimally invasive colorectal surgery with an experience of at least 30 cases of LLC and at least 30 cases of colon and/or rectal robotic resections.

### Matching and statistical analysis

To minimize the impact of treatment allocation bias, RLC patients were matched to LLC patients using propensity scores. Multivariable logistic regression was performed to estimate the propensity to undergo RLC for all patients, regardless of the actual treatment received according to the principles suggested by Austin et al. [13]. Propensity scores were based on baseline variables age, sex, BMI, ASA score, CCI, the presence of malignancy, T4 or complicated diverticular disease, bridge to surgery procedures, and the inclusion in an enhanced recovery protocol. Nearest neighbor matching was performed in a 1:1 ratio without replacement and a caliper width of 0.2 standard deviation (SD) was specified.

Before matching, categorical data were shown as frequencies with percentages and were compared with the Fisher’s exact test, as appropriate. Median and interquartile (IQR) range were used to present continuous variables which were compared with the Mann–Whitney *U* test. After matching, categorical variables were compared with the McNemar’s test while the Wilcoxon signed-rank test was used with continuous data.

## Results

### Total cohort

A total of 300 patients (143 RLC vs 157 LLC) were included in the study. Patients’ preoperative characteristics are shown in Table 1. The two groups significantly differed in terms of ASA score (ASA > 2, RLC 23, 16.1% vs LLC 46, 29.3%; *p* = 0.008), colon cancer (RLC 100, 69.9% vs LLC 86, 54.8%; *p* = 0.009), and enhanced recovery protocol (RLC 31, 21.7 vs LLC 54, 34.4; *p* = 0.015). Operative outcomes are presented in Table 2. The rate of conversion to open surgery (12.7% vs 5.6%, *p* = 0.048) and the creation of stoma (12.1% vs. 5.6%, *p* = 0.008) were higher in the LLC group. Median operative time was significantly longer in RLC group [300 (253–340) vs. 245 (184–296), *p* < 0.0001]. Postoperative outcomes are shown in Table 3. Safety parameters were similar between the groups. Oral feeding was performed in the 4th postoperative day in the RLC group versus 3rd postoperative day in the LLC group (< 0.0001).

**Table 1:** Patients’ characteristics

Variables	Robotic (*n* = 143)	Laparoscopic (*n* = 157)	*p*	Robotic (*n* = 119)	Laparoscopic (*n* = 119)	*p*
Age > 65						
Yes	64 (44.7)	82 (52.3)	0.205	52 (43.7)	58 (48.7)	0.396
No	79 (55.3)	75 (47.7)		67 (56.3)	61 (51.3)	
Female—*n* (%)						
Yes	61 (42.6)	82 (52.3)	0.106	55 (46.2)	61 (51.3)	0.446
No	82 (57.4)	75 (47.7)		64 (53.8)	58 (48.7)	
BMI > 30						
Yes	9 (6.3)	18 (11.4)	0.157	7 (5.9)	13 (10.9)	0.336
No	134 (93.7)	139 (88.6)		112 (94.1)	106 (89.1)	
ASA score > 2						
Yes	23 (16.1)	46 (29.3)	0.008	22 (18.5)	26 (21.8)	0.465
No	120 (83.9)	111 (70.7)		97 (81.5)	93 (78.2)	
CCI > 5						
Yes	27 (18.9)	41 (26.1)	0.167	19 (16.0)	28 (23.5)	0.243
No	116 (81.1)	116 (73.9)		100 (84.0)	91 (76.5)	
Colon cancer						
Yes	100 (69.9)	86 (54.7)	0.009	83 (69.7)	74 (62.2)	0.262
No	43 (30.1)	71 (45.3)		36 (30.2)	45 (37.8)	
Complicated diverticular disease						
Yes	12 (8.4)	22 (14.0)	0.146	9 (7.6)	12 (10.1)	0.512
No	131 (91.6)	135 (86.0)		110 (93.4)	107 (89.9)	
cT4 tumors						
Yes	7 (4.9)	9 (5.7)	0.801	7 (5.9)	6 (5.0)	1.000
No	136 (95.1)	148 (92.3)		112 (94.1)	113 (95.0)	
Bridge procedure to surgery						
Yes	3 (2.1)	10 (6.3)	0.09	2 (16.8)	7 (5.9)	0.125
No	140 (97.9)	147 (93.7)		117 (98.3)	112 (94.1)	
Enhanced recovery protocol						
Yes	31 (21.7)	54 (34.4)	0.015	29 (24.4)	35 (29.4)	0.405
No	112 (78.3)	103 (65.6)		90 (75.6)	84 (70.6)

**Table 2:** Intraoperative variables

Variables	Robotic (*n* = 143)	Laparoscopic (*n* = 157)	*p*	Robotic (*n* = 119)	Laparoscopic (*n* = 119)	*p*
Operative time (min)						
Median (IQR)	300 (253–340)	245 (184–296)	< 0.0001	296 (260–340)	245 (195–296)	< 0.0001
Conversion to open surgery—*n* (%)						
Yes	8 (5.6)	20 (12.7)	0.048	5 (4.2)	9 (7.6)	0.265
No	138 (94.4)	145 (87.3)		114 (75.8)	110 (93.4)	
High tie *n* (%)						
Yes	116 (81.1)	127 (80.9)	0.68	97 (81.5)	100 (84.0)	0.644
No	30 (18.9)	38 (24.2)		21 (18.5)	19 (16.0)	
Stoma						
Yes	4 (2.8)	19 (12.1)	0.008	3 (2.5)	7 (5.9)	0.289
No	142 (97.2)	138 (87.9)		116 (97.5)	112 (94.1)	
Additional resection—*n* (%)						
Yes	19 (13.3)	20 (12.7)	0.864	17 (14.3)	17 (14.3)	1.000
No	127 (86.7)	145 (92.3)		102 (85.7)	102 (85.7)	
Number of lymphnode harvested(min)—median—(IQR)	20 (14–26)	20 (15–26)	0.406	20 (14–26)	20 (14–26)	0.839

**Table 3:** Postoperative variables

Variables	Robotic (*n* = 143)	Laparoscopic (*n* = 157)	*p*	Robotic (*n* = 119)	Laparoscopic (*n* = 119)	*p*
Postoperative complications—*n* (%)						
Yes	33 (23.1)	25	0.143	19 (16.0)	15 (12.6)	0.265
No	110 (76.9)	132		100 (84.0)	104 (87.4)	
Postoperative mortality—*n* (%)						
Yes	1 (0.7)	1 (0.6)	1.000	0 (0.0)	0 (0.0)	1.000
No	142 (99.3)	156 (99.4)		119 (100)	119 (100)	
Postoperative complications Clavien–Dindo > 2—*n* (%)						
Yes	5 (3.6)	5 (3.2)	1.000	0 (0.8)	0 (0.0)	0.572
No	138 (96.4)	152 (96.8)		119 (100)	119 (100)	
Transfusions *n* (%)						
Yes	1 (0.7)	7 (4.4)	0.071	1 (0.8)	4 (3.8)	0.375
No	142 (99.3)	150 (95.6)		118 (99.2)	105 (88.2)	
Re-admissions ≤ 30 days—*n* (%)						
Yes	3 (2.1)	2 (1.2)	0.672	3 (2.5)	2 (1.7)	1.000
No	140 (97.9)	155 (98.8)		116 (97.5)	117 (98.3)	
Time to first flatus—days—median—(IQR)	2 (2–3)	2 (2–3)	0.452	2 (2–3)	2 (2–3)	0.672
Oral feeding—days—(IQR)	4 (3–5)	3 (2–4)	< 0.0001	2 (2–3)	2 (2–4)	0.356
Drain placement—*n* (%)						
Yes	135 (94.4)	146 (94.3)	1.000	112 (94.1)	114 (95.8)	0.754
No	8 (5.6)	9 (5.7)		7 (5.9)	5 (4.2)	
Drain removal—*n* (%)	5 (4–6)	5 (4–6)	0.298	5 (4–6)	5 (4–6)	0.942
Hospital stay—days—median—(IQR)	7 (7–9)	7 (5–8)	0.062	7 (7–8)	7 (5–8)	0.088

## Matched cohort

After matching, there were no significant differences in the variables included in the propensity score (Table 1). Operative time was longer in the RLC group [296 (260–340) vs. 245 (195–296), *p* < 0.0001]. The remaining intraoperative variables were similar between the groups (Table 2), including conversion to open approach (RLC, *n* = 5, 4.2 vs. LLC *n* = 9, 7.6%; *p* = 0.265).

Postoperative complications, mortality, and functional outcomes did not significantly differ between the groups (Table 3). No major complications were found in both groups.

## Discussion

RLC has safety parameters similar to LLC. This was only partially in line with what has already been presented by a recent meta-analysis [6]: as such, the pooled results showed a significant difference in terms of overall postoperative complications favoring the robotic group. This difference was less evident in the pooled analysis of the Clavien–Dindo > 2 for which *p* value was borderline significant (*p* = 0.055). However, it should be noted that only two out [7, 8] of 10 studies, which weighted more than 95% of the pooled cohort, reported improved safety parameters in the robotic group. However, both studies were from national multi-institutional databases and they lacked from operative details (e.g., learning curve, technical details linked to the procedure…). This could have resulted in a difficult interpretation of the published results.

It remains that our results suggested that RLC is safe and feasible and it could be used for training as a propaedeutic step for rectal resections. This was also suggested by Gass et al. [14] who found no differences in terms of safety parameters between the two groups.

Interestingly, our results found no advantages in the conversion rates using the robotic approach. There are conflicting results with regard to this variable. In the above cited meta-analysis [6], the three studies with the largest sample size were the only ones showing benefits in terms of conversion rates associated with the use of the robotic platform [7, 8, 14]. Interestingly, in addition to the two studies based on the two national databases [7, 8], the study by Gass et al. [14] also showed clear advantages with conversion rates in the robotic group, and this was particularly evident in the learning curve adjusted propensity score matched analysis. However, it must be highlighted that the authors based the propensity score on only on demographics (age, sex, and BMI), the indication for surgery (benign versus malignant), and the version of the Da Vinci used. These parameters may have not balanced the two groups for the complex left colectomies performed for some challenging cases of diverticular disease. In order to avoid this bias, we based the propensity score on T4 or complicated diverticular disease, bridge to surgery procedures, and the inclusion in an enhanced recovery protocol, and we found no differences in conversion rates in the post-matched analysis. Still, we sought that the robotic approach may positively influence the conversion rate in specific subgroups, such as complicated diverticular disease and T4 tumors. In this case, the accurate dissection guaranteed by the robotic platform might be able to help the surgeon to avert conversions. No data on these cases are available, and large multicenter studies prospective studies are warranted in order to confirm this hypothesis.

Operative time was longer in the robotic group in RLC as it is in most all robotic procedure in general surgery [2, 15–18]. This may be due to the docking time, which is absent in laparoscopic procedures, and, as it has already been highlighted elsewhere [19], to the different approach to dissection. The robotic platform with its higher dexterity and more accurate view of the operating field may lead to such a meticulous dissection of tissues, which may likely result in a prolonged operative time.

This study has some limitations linked to its retrospective nature. First, the study interval of control group was shorter and more recent and this might have affected the outcomes. The impact of this choice might have been mitigated by propensity score matching. However, it must be taken into consideration when interpreting the results. Second, there were no sufficient data to perform a sub-analysis of specific conditions, such as T4 tumors and complicated diverticular disease, as in our opinion, they may highlight some advantages in using the robotic platform.

In conclusion, RLC have safety parameters and conversion rates which are comparable with standard LLC. In light of this, the robotic approach for left colectomies may be used for training purposes. In addition, further studies on specific indications should be performed in order to find potential clear advantages on the use of the robotic approach also for left colectomies.

## Data Availability

The data that support the findings of this study are available on reasonable request from the corresponding author, [LS].
